# Brown’s Tumor of the Mandible Secondary to Hyperparathyroidism

**DOI:** 10.7759/cureus.98633

**Published:** 2025-12-07

**Authors:** Katerina Vlachou, Shoney Bivin, Richard Burnham

**Affiliations:** 1 Oral and Maxillofacial Surgery, Manchester Royal Infirmary, Manchester, GBR; 2 Oral and Maxillofacial Surgery, Royal Stoke University Hospital, Stoke-on-Trent, GBR

**Keywords:** brown’s tumour, mandible bone tumour, oral manifestation, primary hyperparathyroidism (phpt), surgical case reports

## Abstract

Brown’s tumor (osteitis fibrosa cystica) is a rare, non-neoplastic condition resulting from hyperparathyroidism (HPT). It is characterized by increased osteoclastic activity and bone turnover, which subsequently leads to the deposition of fibrous tissue. Although it generally affects long bones of the body, it can also affect facial bones, particularly the mandible. It can further mimic other fibro-osseous, infective, or malignant conditions. We report the case of a 67-year-old female patient with primary HPT. The initial swelling was found incidentally during an extraction. Radiographic imaging revealed root resorption of the adjacent teeth, while histological examination demonstrated the typical multinucleated giant cells. Elevated serum calcium and parathyroid hormone (PTH) levels confirmed the diagnosis of primary HPT and supported the clinical impression of a Brown's tumor. Brown's tumor is often a diagnostic challenge as it is misdiagnosed, particularly as a malignant lesion. Early detection is crucial in order to provide the appropriate treatment, not only for the lesions but also for the underlying HPT.

## Introduction

Brown’s tumor (osteitis fibrosa cystica) is a benign lesion in the bones resulting from a high osteoclast turnover rate, secondary to hyperparathyroidism (HPT). Excessive production of the parathyroid hormone (PTH) disrupts calcium homeostasis. In bones, specifically, PTH stimulates the action of osteoclasts, resulting in the increased rate of resorption of the matrix, increasing the amount of free calcium ions in the serum. The lesions tend to resolve by treating the underlying HPT [1].

PTH has a key role in regulating calcium homeostasis through its effects on both the renal and skeletal systems. PTH acts on the kidneys as a calcitonin antagonist by stimulating calcium reabsorption and inhibiting phosphate reabsorption. In the skeletal system, it stimulates osteoclast activity to release calcium ions into the circulation. Primary HPT arises from autonomous increased production of PTH from the parathyroid gland, with the most common cause being benign adenomas. Secondary HPT is a compensatory mechanism due to systemic calcium dysregulation. Hypocalcemia caused by conditions such as chronic kidney disease and vitamin D deficiency increases the production of PTH. The compensatory mechanism results in increased bone turnover [1].

Brown’s tumors secondary to HPT are relatively rare, with incidence rates around 2%-5% within the population affected by the condition. It is characterized by the proliferation of giant cell granulomas and most often affects the phalanges, clavicles, ribs, and pelvic girdle, but can, in some rarer cases, affect craniofacial bones [2]. Our case reports the development of Brown’s tumor on the anterior mandible region.

This article was previously presented as a poster at the JTG (Junior Trainees Group of BAOMS) Conference 2025 on November 8, 2025. This article was previously posted to the SSRN preprint server on September 30, 2025.

## Case presentation

**Figure 1:**
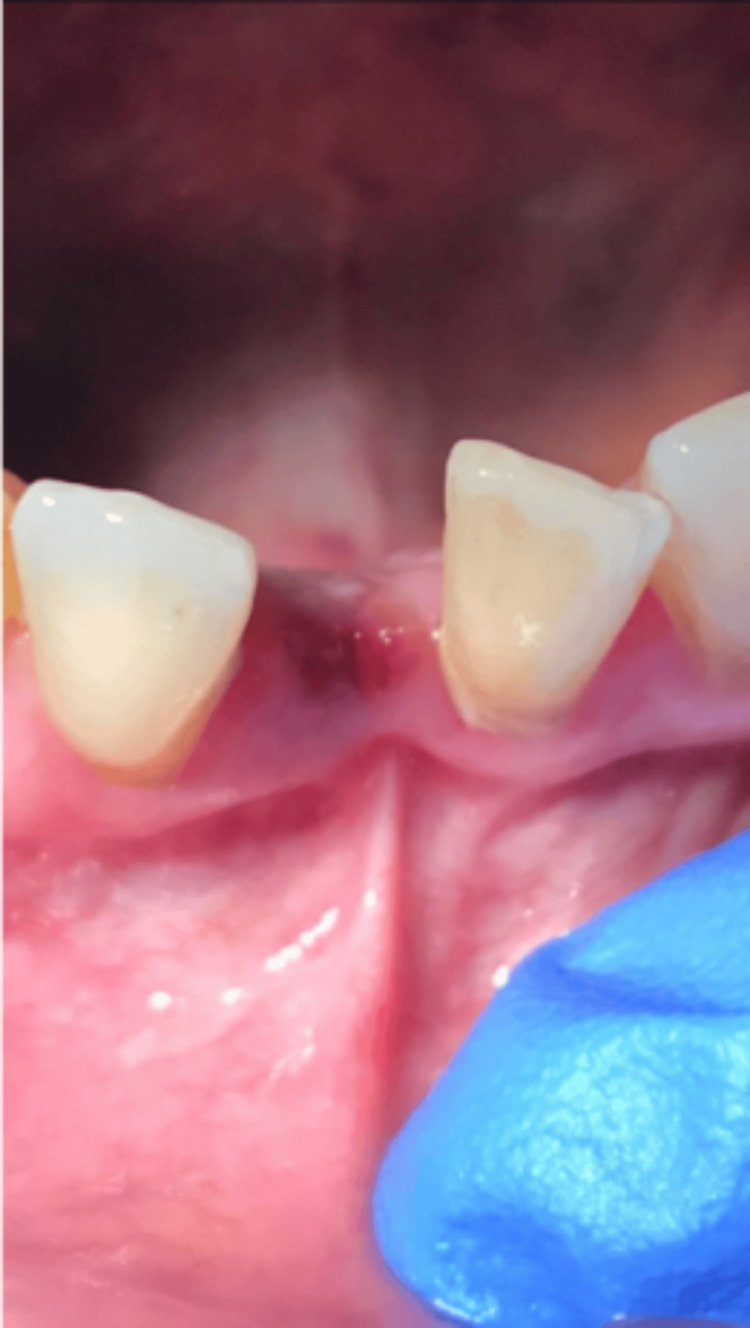
Intraoral photograph of the anterior mandibular lesion The lesion appears as a blue-purple swelling on the lower anterior mandible following the extraction of the lower right central incisor.

**Figure 2:**
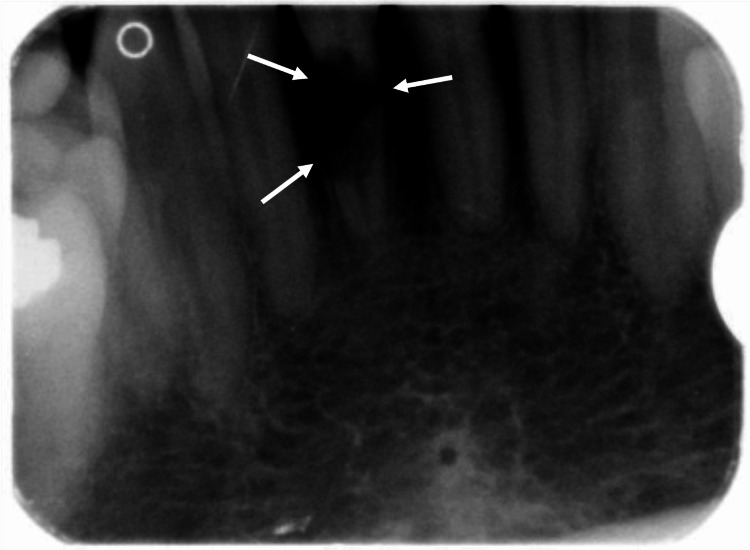
Periapical radiograph demonstrating extensive root resorption of the lower right central incisor prior to extraction.

A 67-year-old woman was referred to the oral and maxillofacial surgery department for a raised blue-purple lesion on the lower alveolar ridge (Figure 1). The swelling was found incidentally following dental extraction of the lower right central incisor due to extensive root resorption (Figure 2). The patient was otherwise fit and healthy with no additional symptoms, making the clinical findings incidental.

Excision of the lesion was performed under local anesthesia, and histology showed stromal proliferation of multinucleated giant cells accompanied by a population of spindle cells, hemorrhage, and abundant hemosiderin deposition. The absence of atypia ruled out malignancy. As the clinical and histological presentations in isolation could not point to a definitive diagnosis, blood tests, including a bone profile, were also performed. These findings, in combination with elevated calcium of 2.95 mmol/L (reference range: 2.20 - 2.60 mmol/L) and PTH of 67.64 pmol/L (reference range: 1.6 - 6.9 pmol/L), confirmed the differential diagnosis of Brown’s tumor secondary to primary HPT.

The patient was subsequently referred to the endocrinology and otolaryngology teams for further workup and definitive management with a left parathyroidectomy. Intraoperatively, a large adenoma (45 x 30 x 15 mm) was identified and excised from the left superior parathyroid gland.

The patient remained on outpatient follow-up with endocrinology and otolaryngology teams for one year before discharge and was advised to have annual calcium checks with her general practitioner.

## Discussion

Brown’s tumor is a non-neoplastic, reactive lesion caused by HPT, with primary disease referring to pathology directly affecting the parathyroid gland, secondary disease resulting from chronic kidney disease, and tertiary disease caused by autonomous PTH secretion. Elevated levels of PTH stimulate osteoclast activation, resulting in increased rates of bone turnover. Subsequently, bone is replaced with fibrous tissue, giving rise to the lesions, characterized by the presence of giant cells.

The development of Brown’s tumor is rare, with approximately 2%-5% prevalence in patients with HPT, and even rarer in the craniofacial bones [1]. Although Brown's tumors are slow-growing and sometimes asymptomatic, they have the potential to grow to the extent where they can cause disfigurement [2,3]. Typically, these lesions are characterized as well-defined radiolucencies, which often cause local destruction. In the mandible, this presents as loss of lamina dura, tooth displacement, and root resorption [2,3]. Accurate recognition and diagnosis are crucial, as radiographically, it mimics other conditions. Examples include fibrous dysplasia, giant cell lesions, and aneurysmal bone cysts. Bony malignancies and metastases cannot be excluded either [3].

The distinct histological features include the presence of macrophages, giant multinucleated osteoclasts, and deposits of hemosiderin, which result from local hemorrhage and give the lesion its characteristic appearance and name [4]. As such, the correct diagnosis is dependent on clinical presentation and biochemistry. Blood results will be consistent with HPT and will show hypercalcemia, hypophosphatemia, and increased levels of PTH and alkaline phosphatase [5].

## Conclusions

Brown's tumor can be a diagnostic challenge for clinicians, despite being a relatively rare presentation. As seen in this case, such lesions can mimic other pathologies, including other fibro-osseous conditions or even cancer. Extensive root resorption should raise suspicion and warrant further investigations that go beyond biopsy results. Since the characteristic multinucleated giant cells overlap with those seen in other giant cell lesions, a definitive diagnosis cannot be made from histological findings alone. Therefore, the case should be viewed holistically by pairing tissue findings with biochemical evidence of HPT, including elevated serum calcium and PTH levels. A prompt and accurate definitive diagnosis of Brown's tumor is essential, as it is the direct result of a systemic endocrine pathology. It can therefore allow for the correct management of the underlying parathyroid disorder, which in turn will not only resolve the lesion but also prevent further complications arising from the primary condition. 

## References

[1] Guedes A, Becker RG, Nakagawa SA, Guedes AA (2024). Update on brown tumor of hyperparathyroidism. Rev Assoc Med Bras (1992).

[2] Can Ö, Boynueğri B, Gökçe AM (2016). Brown tumors: a case report and review of the literature. Case Rep Nephrol Dial.

[3] Diacinti D, Cipriani C, Biamonte F (2021). Imaging technologies in the differential diagnosis and follow-up of brown tumor in primary hyperparathyroidism: case report and review of the literature. Bone Rep.

[4] Rosenberg AE, Nielsen GP (2001). Giant cell containing lesions of bone and their differential diagnosis. Curr Diagn Pathol.

[5] Zhu CY, Sturgeon C, Yeh MW (2020). Diagnosis and management of primary hyperparathyroidism. JAMA.

